# JMJD1C promotes smooth muscle cell proliferation by activating glycolysis in pulmonary arterial hypertension

**DOI:** 10.1038/s41420-023-01390-5

**Published:** 2023-03-18

**Authors:** Chen Zhang, Yue Sun, Yingying Guo, Jingjing Xu, Haiyan Zhao

**Affiliations:** 1 Department of Obstetrics and Gynecology, Shengjing Hospital of China Medical University, Shenyang, China; 2 Department of Rheumatology and Immunology, Shengjing Hospital of China Medical University, Shenyang, China

**Keywords:** Mechanisms of disease, Predictive markers

## Abstract

Pulmonary arterial hypertension (PAH) is a chronic disorder characterized by hyperproliferation of pulmonary arterial smooth muscle cells (PASMCs). JMJD1C, a member of the Jumonji domain containing C (JMJC) histone demethylase family, contributes to cardiovascular dysfunction. However, the role of JMJD1C in PAH remains unknown. Mice were exposed to hypoxia to mimic several features associated with PAH clinically. We found that JMJD1C was highly expressed in the lungs of mice after hypoxia exposure. JMJD1C knockdown ameliorated hypoxia-induced right ventricular remodeling and thickening of the pulmonary arterial wall. PASMC hyperproliferation and resistance to apoptosis in mice exposed to hypoxia were suppressed by JMJD1C inhibition. We demonstrated that JMJD1C silencing reduced glycolytic enzymes (HK2, PGK1 and LDHA) and lactate overaccumulation in the lungs of mice exposed to hypoxia. In vitro, hypoxia-induced hyperproliferation and activated glycolytic processes in mouse PASMCs were impaired by JMJD1C knockdown. In addition, the activation of STAT3 signaling by hypoxia was suppressed by JMJD1C silencing both in vivo and in vitro. The overexpression of STAT3 reversed the inhibitory effect of JMJD1C depletion on proliferation and glycolysis in PASMCs under hypoxia. Thus, JMJD1C induces glycolytic processes by activating STAT3 signaling to promote PASMC proliferation and pulmonary vascular remodeling, suggesting the potential role of JMJD1C in regulating the metabolic program and vascular remodeling in PAH.

## Introduction

Pulmonary arterial hypertension (PAH) is a rare and fatal cardiopulmonary disease characterized by a progressive increase in pulmonary vascular resistance and pulmonary vessel remodeling, which ultimately leads to right ventricular failure and death [1]. Vasoconstriction, endothelial cell dysfunction, smooth muscle cell proliferation, extracellular matrix accumulation, and inflammation are important processes associated with PAH [2]. Although current therapies can improve symptoms and hemodynamic parameters in PAH, they do not significantly reduce morbidity and mortality. Thus, novel approaches for treating PAH are urgently needed.

Glycolysis, or the Warburg effect, has been suggested to be a critical pathogenic pathway in the development of PAH [3]. Positron emission tomography shows dramatic glucose uptake in the lungs of idiopathic PAH patients, indicating increased glycolytic activity in the lungs during PAH [4]. In addition, it has been demonstrated that both pulmonary arterial smooth muscle cells (PASMCs) and endothelial cells from patients or animals with PAH undergo significant glycolysis and then hyperproliferate [5]. PASMCs are one of the major cell types that affect vascular remodeling in PAH, and these cells exhibit several pathological features, such as hyperproliferation, resistance to apoptosis and metabolic disturbances [6]. Kovacs et al. suggested that aberrant glycolysis contributed to significant collagen synthesis and proliferation in PASMCs during PAH with vascular remodeling [7]. The underlying mechanisms of glycolysis in PASMCs still need further investigation.

JMJD1C is a member of the Jumonji domain containing C (JMJC) family, and it functions as a histone demethylase to activate gene transcription [8, 9]. For example, Viscarra et al. showed that JMJD1C regulated the lipogenic process by demethylating H3K9 at lipogenic promoters [10]. Chen et al. found that JMJD1C acted as a critical transcriptional coactivator to regulate the survival of acute myeloid leukemia cells [11]. Furthermore, a previous study by Lynch et al. indicated that JMJD1C had an effect on glycolytic and oxidative programs in HOXA9-dependent leukemogenesis [12]. Zhang et al. demonstrated that JMJD1C accelerated angiotensin II-induced cardiac hypertrophy and fibrosis in experimental mouse models [13, 14]. More importantly, microarray analysis showed significantly high expression of JMJD1C in the lungs of PAH patients, suggesting an association between JMJD1C and the risk of PAH [15]. However, the effect of JMJD1C on the development of PAH remains unknown.

In this study, mice or PASMCs were exposed to hypoxia to investigate whether JMJD1C knockdown affected the development of PAH. The glycolytic process was mediated by the STAT3 signaling pathway and was investigated to uncover the underlying mechanism of JMJD1C.

## Results

### JMJD1C expression is increased in the lungs of mice exposed to hypoxia

**Fig. 1:**
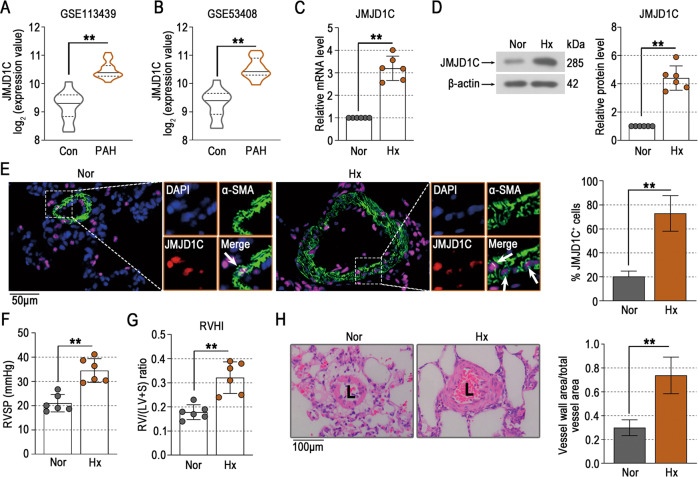
JMJD1C expression is increased in the lungs of mice exposed to hypoxia. **A**, **B** Expression levels of JMJD1C mRNA in lung homogenates of PAH patients in GSE113439 and GSE53408. *N* = 11–15. **C**, **D** Relative mRNA and protein levels of JMJD1C in the lung were examined by qPCR and Western blotting. **E** Representative lung tissue sections were subjected to immunofluorescence staining of α-SMA (green) and JMJD1C (red). DAPI was used to stain cell nuclei (blue). Bar = 50 μm. Arrows indicate the coexpression of α-SMA and JMJD1C in pulmonary arteries. **F**, **G** The RVSP, and RVHI were determined in mice exposed to normoxia or hypoxia. **H** Representative histological images of lung sections stained with hematoxylin and eosin (H&E). The wall thickness of the pulmonary arteries was measured. Bar = 100 μm. Nor normoxia; Hx hypoxia; L Lumen. The values are the mean ± SD, and statistical significance was assessed by unpaired t-test. *N* = 6, **p < 0.01.

The dysregulated genes in the lung homogenates of PAH patients were downregulated, as shown by the Gene Expression Omnibus (GEO) [16] under accession numbers GSE113439 and GSE53408. The microarray data from GSE113439 and GSE53408 showed that the mRNA level of JMJD1C was increased in PAH patients (Fig. 1A, B). Similar alterations in JMJD1C at both the mRNA and protein levels were observed in the lungs of mice exposed to hypoxia (Fig. 1C, D). The immunofluorescence staining results suggested a high expression level of JMJD1C in the smooth muscle cells of mice exposed to hypoxia (Fig. 1E). Right ventricular remodeling was observed in mice exposed to hypoxia, as evidenced by increases in the right ventricular systolic pressure (RVSP) and right ventricular hypertrophy index (RVHI) (Fig. 1F, G). The thickness of the pulmonary arterial wall was also increased by hypoxia, as shown by HE staining **(**Fig. 1H**)**. This finding suggests that high expression of JMJD1C may participate in the development of pulmonary hypertension.

### JMJD1C knockdown attenuates pathological changes in mice exposed to hypoxia

**Fig. 2:**
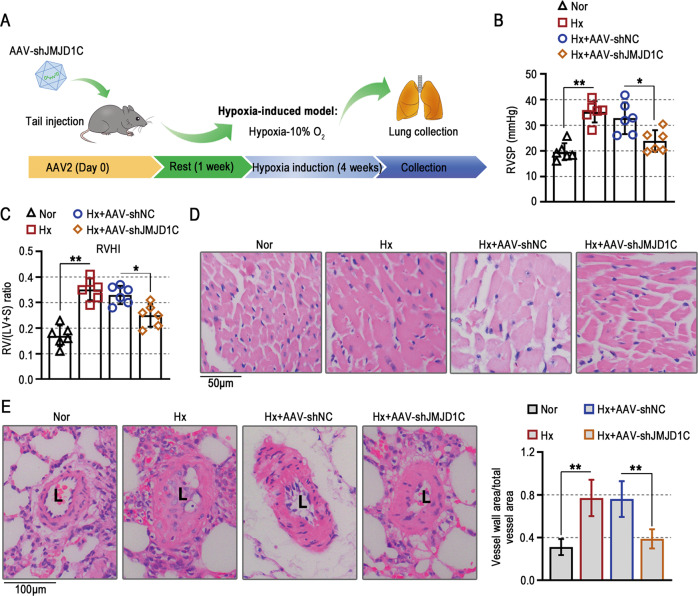
JMJD1C knockdown attenuates pathological changes in mice exposed to hypoxia. **A** Illustration of the animal protocols. **B**, **C** The RVSP and RVHI in mice exposed to normoxia or hypoxia were examined. **D** Representative histological images of heart sections stained with H&E. Bar = 50 μm. **E** Representative histological images of lung sections stained with H&E. The wall thickness of the pulmonary arteries was measured. Bar = 100 μm. Nor Normoxia, Hx Hypoxia, L Lumen. The values are the mean ± SD, and statistical significance was determined by one-way ANOVA. *N* = 6, *p  < 0.05, **p < 0.01.

AAV2 vectors for JMJD1C knockdown were used to study its role in pulmonary hypertension, and the protocol timeline is shown in Fig. 2A. We demonstrated that JMJD1C knockdown inhibited hypoxia-induced increases in RVSP and RVHI (Fig. 2B, C). Right ventricular hypertrophy and pulmonary arterial wall thickening in mice exposed to hypoxia were attenuated by JMJD1C inhibition (Fig. 2D, E). Taken together, these results suggest that JMJD1C contributes to the development of right ventricular hypertrophy and pulmonary arterial remodeling in response to hypoxia exposure.

### JMJD1C knockdown decreases PASMC hyperproliferation and resistance to apoptosis in mice exposed to hypoxia

**Fig. 3:**
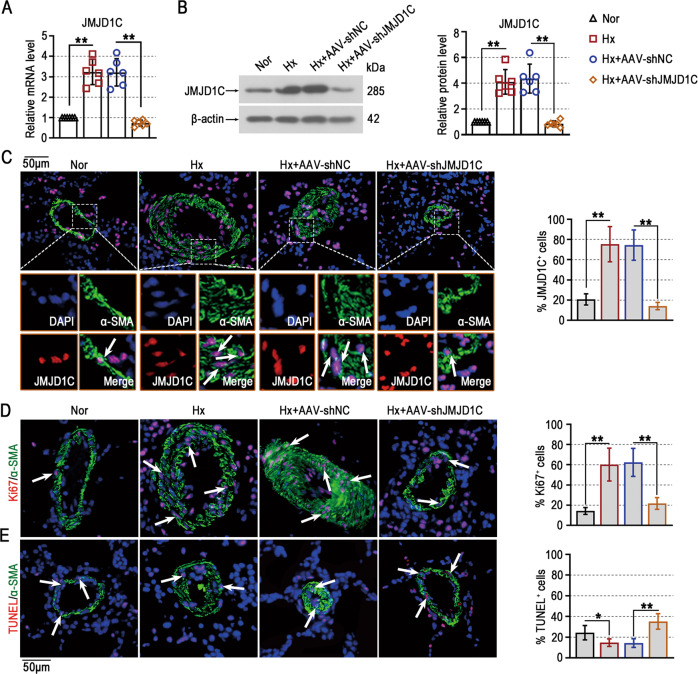
JMJD1C knockdown decreases PASMC hyperproliferation and resistance to apoptosis in mice exposed to hypoxia. **A**, **B** The mRNA and protein levels of JMJD1C in lungs were detected using qPCR and Western blotting. **C** Representative lung sections were subjected to immunofluorescence staining of α-SMA (green) and JMJD1C (red) (left panel). DAPI was used to stain cell nuclei (blue). Arrows indicate the coexpression of α-SMA and JMJD1C in pulmonary arteries. The percentage of JMJD1C^+^ cells in pulmonary arteries (stained with α-SMA) was quantified (right panel). Bar = 50 μm. **D** Representative immunofluorescence images of lung sections stained with anti-α-SMA antibodies (green) and anti-Ki67 antibodies (red) (left panel). DAPI was used to stain cell nuclei (blue). Arrows indicate the colocalization of α-SMA and Ki67 in pulmonary arteries. The percentage of Ki67^+^ cells in pulmonary arteries (stained with α-SMA) was quantified (right panel). Bar = 50 μm. **E** Representative immunofluorescence images of lung sections stained with anti-α-SMA antibodies (green) and TUNEL (red) (left panel). DAPI was used to stain cell nuclei (blue). Arrows indicate the colocalization of α-SMA and TUNEL in pulmonary arteries. The percentage of TUNEL^+^ cells in pulmonary arteries (stained with α-SMA) was quantified (right panel). Bar = 50 μm. Nor Normoxia, Hx Hypoxia. The values are the mean ± SD, and statistical significance was determined by one-way ANOVA. *N* = 6, *p < 0.05, **p < 0.01.

For the JMJD1C knockdown studies, we found that the mRNA and protein levels of JMJD1C in the lungs of mice exposed to hypoxia were reduced by AAV administration (Fig. 3A, B). The results of JMJD1C^+^-α-SMA^+^ immunofluorescence staining showed that JMJD1C knockdown inhibited hypoxia-induced JMJD1C enhancement in the PASMCs of mice (Fig. 3C). This finding indicates the potential role of JMJD1C in the changes in PASMCs during PAH.

PASMC hyperproliferation and resistance to apoptosis are key events during PAH development. The results of Ki67 staining showed that hypoxia-induced PASMC hyperproliferation was inhibited by JMJD1C depletion in vivo (Fig. 3D). The hypoxia-induced reduction in TUNEL-positive PASMCs was increased by JMJD1C knockdown (Fig. 3E). Taken together, these data suggest that JMJD1C promotes PASMC hyperproliferation and resistance to apoptosis in mice exposed to hypoxia.

### JMJD1C knockdown prevents glycolysis in mice exposed to hypoxia

**Fig. 4:**
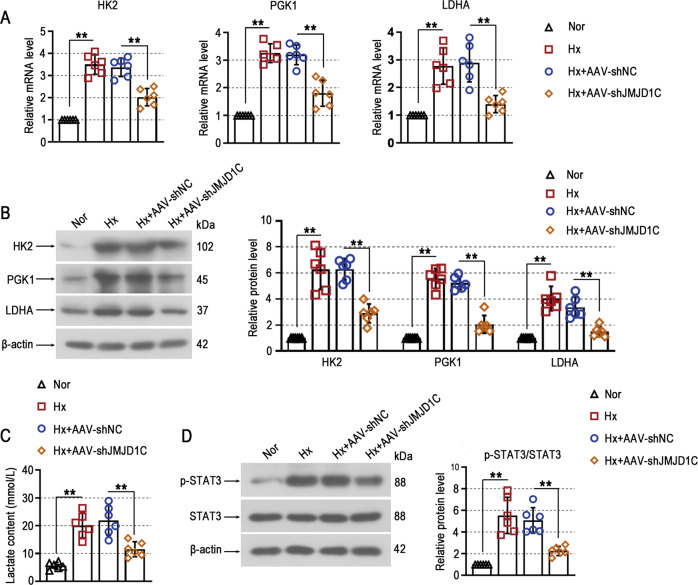
JMJD1C knockdown prevents glycolysis in mice exposed to hypoxia. **A** The mRNA levels of HK2, PGK1 and LDHA in lung tissues were measured by qPCR. **B** Western blot analysis of HK2, PGK1 and LDHA protein expression in lung tissues. **C** Serum lactate levels were measured. **D** The protein levels of p-STAT3 and STAT3 were examined using Western blotting. Nor Normoxia, Hx Hypoxia. The values are the mean ± SD, and statistical significance was determined by one-way ANOVA. *N* = 6, **p < 0.01.

Glycolysis is an important step in the production of ATP and lactate, and it drives the hyperproliferation of PASMCs during pulmonary hypertension development. We examined the expression of glycolytic enzymes, including hexokinase II (HK2), phosphoglycerate kinase 1 (PGK1) and lactate dehydrogenase A (LDHA), to evaluate the effect of JMJD1C on PAH. The results showed that the hypoxia-induced increase in glycolytic enzymes at the mRNA and protein levels in lung homogenates was reduced by JMJD1C knockdown (Fig. 4A, B). Lactate is an end product of glycolysis, and we demonstrated that JMJD1C silencing inhibited hypoxia-induced lactate overaccumulation in the serum of mice (Fig. 4C). In addition, the increase in the phosphorylation of STAT3 in the lungs of mice exposed to hypoxia was prevented by JMJD1C knockdown (Fig. 4D). These results suggest that JMJD1C enhances glycolysis in hypoxia-induced pulmonary hypertension.

### JMJD1C expression is upregulated in PASMCs exposed to hypoxia

**Fig. 5:**
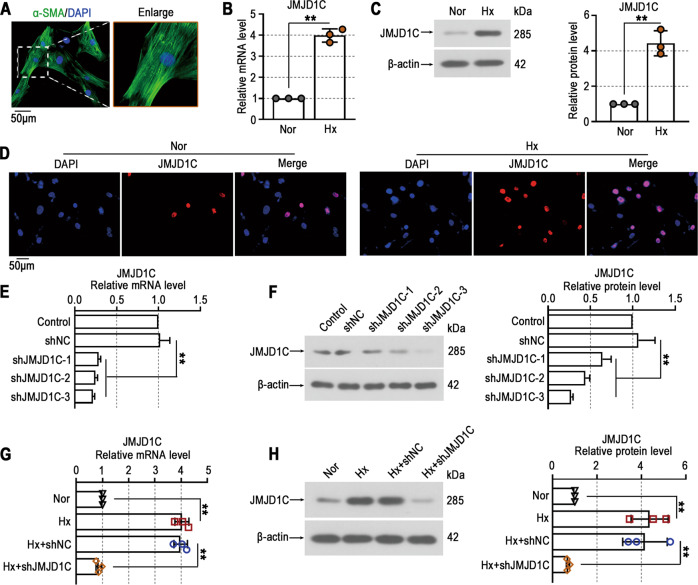
JMJD1C expression is upregulated in PASMCs exposed to hypoxia. **A** Immunofluorescence staining with anti-α-SMA antibodies (green) was performed to determine the purity of PASMCs. Cell nuclei were stained with DAPI (blue). Bar = 50 μm. **B** The mRNA levels of JMJD1C in PASMCs were assessed by qPCR. **C** Western blotting was performed to examine JMJD1C protein expression in PASMCs. **D** Representative immunofluorescence images of PASMCs stained with anti-JMJD1C antibodies (red). Cell nuclei were stained with DAPI (blue). Bar = 50 μm. **E**, **G** qPCR analysis of JMJD1C expression in PASMCs. **F**, **H** Western blot analysis of JMJD1C protein levels in PASMCs. Nor normoxia, Hx Hypoxia. The values are the mean ± SD, and statistical significance was determined by unpaired t-test (**B**, **C**) or one-way ANOVA (**E**–**H**). *N* = 3, **p < 0.01.

Primary PASMCs were used to investigate the function of JMJD1C knockdown in vitro. The purity of isolated PASMCs was identified using an α-SMA antibody by immunofluorescence staining (Fig. 5A). To mimic the condition of PASMCs in vivo, PASMCs were exposed to normoxia or hypoxia (1% O_2_) for 48 h. The results showed that the expression of JMJD1C was upregulated in PASMCs exposed to hypoxia (Fig. 5B, C). Similar alterations in JMJD1C expression were observed in PASMCs by immunofluorescence staining (Fig. 5D). Then, three pairs of shRNAs targeting JMJD1C were designed to inhibit its expression in vitro (Fig. 5E, F). The hypoxia-induced high expression of JMJD1C in PASMCs was downregulated by the shRNA (Fig. 5G, H). The in vitro results show the potential importance of JMJD1C in PASMCs.

### JMJD1C knockdown prevents proliferation and glycolysis in PASMCs exposed to hypoxia

**Fig. 6:**
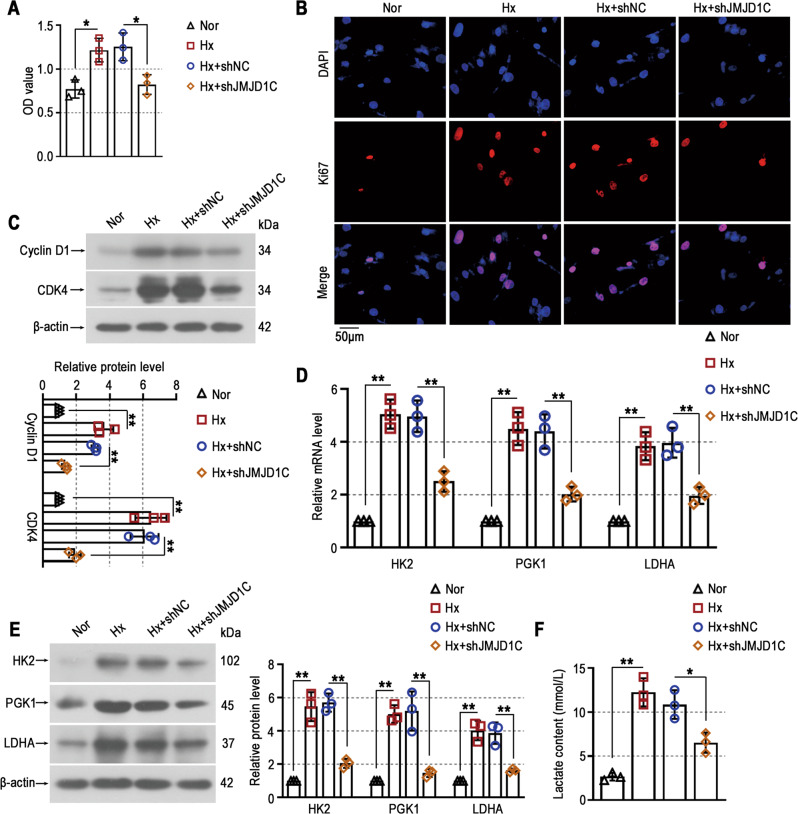
JMJD1C knockdown prevents proliferation and glycolysis in PASMCs exposed to hypoxia. **A** The survival of PASMCs was assessed by CCK8 assays. **B** Representative immunofluorescence images of PASMCs stained with anti-Ki67 antibodies (red). Cell nuclei were stained with DAPI (blue). Bar = 50 μm. **C** Western blot analysis of Cyclin D1 and CDK4 protein levels in PASMCs. **D** The mRNA levels of HK2, PGK1, and LDHA in PASMCs were determined by qPCR. **E** Western blot analysis of HK2, PGK1, and LDHA protein levels in PASMCs. **F** Cell supernatants were used to assess lactate levels. Nor Normoxia, Hx Hypoxia. The values are the mean ± SD, and statistical significance was determined by one-way ANOVA. *N* = 3, *p < 0.05, **p < 0.01.

Then, the effect of JMJD1C in vitro was examined. We found that JMJD1C knockdown reduced hypoxia-induced high levels of PASMC survival by CCK8 assays (Fig. 6A). The results of Ki67 immunofluorescence staining showed that the hyperproliferation of PASMCs exposed to hypoxia was inhibited by JMJD1C silencing (Fig. 6B). The increases in the protein levels of Cyclin D1 and CDK4 in PASMCs exposed to hypoxia were downregulated by JMJD1C knockdown (Fig. 6C). Similar to the in vivo results, the hypoxia-induced increases in glycolytic enzymes and production of lactate were reduced by JMJD1C inhibition in vitro (Fig. 6D–F). Taken together, the in vitro results suggest that JMJD1C facilitates hyperproliferation and glycolytic processes in hypoxia-induced PASMCs.

### STAT3 signaling activation is regulated by JMJD1C and stimulates PASMC proliferation and glycolysis

**Fig. 7:**
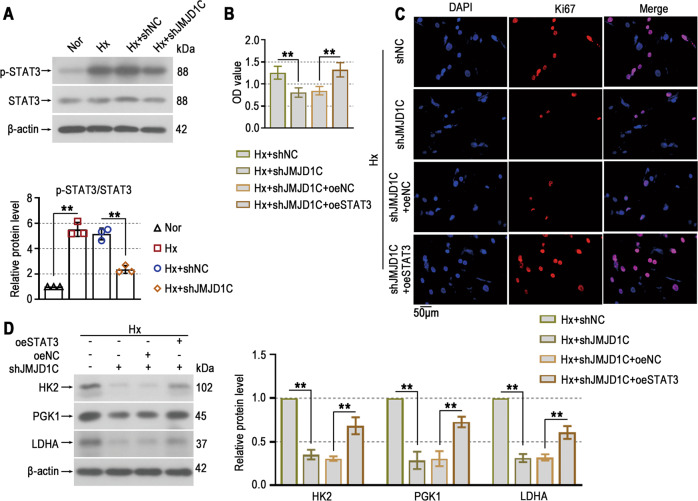
STAT3 signaling activation is regulated by JMJD1C and stimulates PASMC proliferation and glycolysis. **A** Protein levels of p-STAT3 and STAT3 in PASMCs were determined by Western blotting. **B** The CCK8 assay was used to examine the survival of PASMCs. **C** Representative immunofluorescence images of PASMCs stained with anti-Ki67 antibodies (red). Cell nuclei were stained with DAPI (blue). Bar = 50 μm. **D** Western blotting was performed to detect the protein levels of HK2, PGK1, and LDHA in PASMCs. Nor Normoxia, Hx Hypoxia. The values are the mean ± SD, and statistical significance was determined by one-way ANOVA. *N* = 3, **p < 0.01.

Consistent with the in vivo results, we demonstrated that knockdown of JMJD1C inactivated STAT3 signaling in PASMCs exposed to hypoxia (Fig. 7A). The STAT3 overexpression plasmid was used to explore the underlying mechanism. The results showed that the inhibitory effect of JMJD1C on survival in hypoxia-induced PASMCs was reversed by STAT3 (Fig. 7B). STAT3 overexpression enhanced the reduction in Ki67^+^ cells in JMJD1C-knockdown cells (Fig. 7C). In addition, the JMJD1C knockdown-induced increase in the expression of glycolytic enzymes was reversed by STAT3 (Fig. 7D). Taken together, these results suggest that JMJD1C activates STAT3 signaling to promote hyperproliferation and glycolytic abnormalities in PASMCs exposed to hypoxia.

## Discussion

In this study, we demonstrate that high JMJD1C expression promotes pulmonary arterial vascular remodeling and right ventricular hypertrophy in mice exposed to hypoxia. Hypoxia-induced PASMC hyperproliferation and resistance to apoptosis were inhibited by JMJD1C knockdown. The aberrant glycolytic processes in response to hypoxia mediate the effect of JMJD1C on the development of pulmonary hypertension by activating STAT3 signaling.

It is well documented that a variety of regulatory genes and environmental factors cause the development of PAH, which has complicated pathological mechanisms [17]. Hypoxia is a principal biological phenomenon that exerts significant effects on cell physiology [18], and it has been shown to be correlated with cellular phenotypes, such as proliferation, survival, inflammation and metabolic abnormalities [19–21]. In fact, hypoxia-associated pathways act as critical drivers of various diseases, including cancer, stroke, and PAH [22–24]. Our study showed that significant pathological features associated with PAH in the clinic were observed in mice exposed to hypoxia, as evidenced by pulmonary vascular remodeling and right ventricular hypertrophy. Previous studies demonstrated that JMJD1C promoted the development of cardiac hypertrophy in angiotensin II-induced mice [13, 14]. In this study, we found that JMJD1C expression was upregulated in mice exposed to hypoxia, and JMJD1C knockdown attenuated the development of pulmonary hypertension.

PASMCs in PAH were reported to become hyperproliferative and resistant to apoptosis, sharing similar features with cancer cells [19]. Liu et al. suggested that inhibiting the proliferation and migration of PASMCs protected against PAH [25]. It has been documented that JMJD1C drives the proliferation, migration, and invasion in a variety of cancers [26–28]. In agreement with these previous studies in cancers, we observed the proliferative and antiapoptotic role of JMJD1C in PASMCs exposed to hypoxia. However, several contradictory studies have shown an antiproliferative effect of JMJD1C on cancer cells. For example, Zhong et al. suggested that JMJD1C blocked cell growth by promoting M1 macrophage polarization in glioma [29]. We thought that the effect of JMJD1C on cell proliferation might be attributed to different pathological states.

Glycolysis is an important metabolic pathway that stimulates ATP production by consuming glucose [30]. Previous studies indicated that a metabolic shift toward glycolysis was observed during the development of PAH [31, 32]. Werle et al. suggested that the regulation of glycolysis controlled the proliferation of vascular smooth muscle cells [33]. In hypoxia-induced pulmonary hypertension, the metabolic shift toward glycolysis was reported to drive a hyperproliferative phenotype in PASMCs [34]. Kovacs et al. stated that PFKFB3, a key glycolytic regulator, promoted cell proliferation and vascular remodeling by activating glycolytic metabolism in PAH [7]. In this study, the positive regulatory effect of JMJD1C on glycolysis was observed in hypoxia-induced pulmonary hypertension, which was in accordance with that described in acute myeloid leukemia cells [12].

The STAT3 signaling pathway controls pulmonary arterial remodeling in PAH. The inflammatory cytokine IL-6 causes STAT3 activation, which correlates with excessive proliferation and apoptotic resistance in PASMCs [35]. Yerabolu et al. proposed that blocking STAT3-mediated signaling pathways attenuated pulmonary vascular remodeling in experimental PAH models [36]. It was discovered that constitutively active STAT3 was also essential for accelerating glycolysis [37]. Our results showed that STAT3 signaling activation was a potential pathway that mediated the role of JMJD1C in PASMC hyperproliferation and glycolysis. In addition, extracellular matrix (ECM) remodeling and pulmonary arterial wall thickening are the major features of PAH, and they suppress arterial compliance or increase vascular stiffness [38]. The increased production of matrix metalloproteinases could degrade ECM components and promote vascular smooth muscle cell proliferation and migration [39, 40]. IL-6-mediated STAT3 signaling activation was reported to be involved in ECM deposition [41]. Thus, the activation of STAT3 signaling might be induced by IL-6 to regulate ECM remodeling, ultimately contributing to hyperproliferation, apoptosis resistance and glycolysis in PAH.

**Fig. 8:**
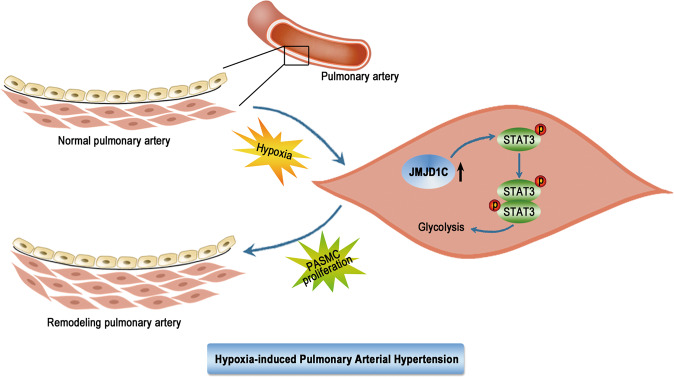
Schematic illustration of the role of JMJD1C in hypoxia-induced PAH. PASMC Pulmonary arterial smooth muscle cell.

In conclusion, the present study shows that high JMJD1C expression contributes to hypoxia-induced vascular remodeling and right ventricular remodeling. The activation of the STAT3 signaling pathway is regulated by JMJD1C and results in a significant proliferative phenotype in PASMCs through the enhancement of glycolysis (Fig. 8). This study provides a novel molecular target for developing new therapies for PAH.

## Materials and methods

### Animal models

Animal protocols were approved by the Ethics Committee of Shengjing Hospital of China Medical University (No. 2019PS106K) and were carried out according to the Guide for the Care and Use of Laboratory Animals. Male C57BL/6 mice (8 weeks; HuaFuKang, Beijing, China) were randomly exposed to normoxia (21% O_2_) or hypoxia (10% O_2_ and 90% N_2_) for 4 weeks as previously described [42]. Briefly, the mice (*n* = 72) were housed in a chamber (ProOx-100HE, TOW-INT TECH, Shanghai, China) with a mixture of oxygen and nitrogen. The oxygen concentration was monitored and controlled by the ProOx-100HE chamber. Soda-lime granules were used to remove CO_2_. All mice were alive and finished the experimental protocols in this study. For the JMJD1C knockdown studies, the synthesized JMJD1C shRNA was cloned into the adeno-associated virus serotype 2 (AAV2) vector. The mice were pretreated with AAV-shJMJD1C or AAV-shNC at a dose of 1.0 × 10^10^ vg/mouse for one week via tail vain injection.

### Analysis of RVSP and RVHI

RVSP and RV hypertrophy were measured to determine the development of pulmonary hypertension. The right cardiac catheterization technique was performed to measure RVSP. In brief, a polyethylene catheter (PE10, Smiths Medical, London, UK) was inserted into the right ventricle through the right jugular vein and connected to a pressure transducer. All data were acquired by a BL420S Biological Signaling Recording and Analysis System (TechMan, Chengdu, China). After the RVSP was measured, the mice were euthanized, and blood, lung and heart were harvested. The RV, left ventricle (LV) and septum (S) were dissected and weighed to calculate the ratio of RV/(LV + S) as an indicator of RVHI.

### Histological analysis

To examine the histological changes in pulmonary arteries and RV hypertrophy, fixed lung or heart tissues were embedded in paraffin and sectioned into 5 μm slices. Then, hematoxylin (H8070, Solarbio, Beijing, China) and eosin (A600190, Sangon, Shanghai, China) were used to stain the sections. Images were captured with an Olympus BX53 microscope (Olympus, Tokyo, Japan) at × 200 or × 400 magnification. To quantify vascular wall thickness, the total vascular area and lumen area were measured blindly, and the ratio of the total vascular area-lumen area to the total vascular area was calculated.

### Harvesting mouse PASMCs

Primary mouse PASMCs were isolated as previously described [42]. Briefly, lung tissues were perfused with PBS, dissected into small pieces and digested in PBS containing collagenase type 2 for 30 min at 37 °C. After being centrifuged for 5 min at 4 °C, the resuspended cells were cultured in DMEM (G4510, Servicebio, Wuhan, China) containing 10% FBS in a humidified incubator at 37 °C with 5% CO_2_. Immunofluorescent staining was performed to determine the purity of the isolated PASMCs by using α-SMA antibodies. Cells at passages 4–7 were prepared for further experiments. Primary PASMCs were confirmed to be negative for mycoplasma.

### Cell culture and treatment

After being cultured in serum-free medium for 24 h, PASMCs were exposed to normoxia (21% O_2_) or hypoxia (1% O_2_) for 48 h. The constructed plasmid expressing JMJD1C shRNA (shJMJD1C) or empty vector (shNC) was transfected into PASMCs for 24 h to knockdown JMJD1C expression. To examine the function of STAT3 signaling, cells were transfected with a plasmid overexpressing STAT3 (oeSTAT3) for 24 h.

### CCK8 assay

To examine cell proliferation, PASMCs were seeded in 96-well plates and cultured in DMEM containing 10% FBS. Then, the cells were incubated with CCK8 solution (KGA317, KeyGEN, Nanjing, China) for 2 h, and the OD value was measured using a microplate reader (800Ts, BIOTEK, Winooski, VT, USA) at 450 nm.

### Immunofluorescence staining

Double immunofluorescence staining was performed to detect the expression of JMJD1C and Ki67 in pulmonary arteries. The paraffin-embedded slices were incubated with primary antibodies against JMJD1C (1:100; NBP1-77072, Novus Biologicals, Littleton, CO, USA), Ki67 (1:100; AF0198, Affinity, Changzhou, China), or α-SMA (1:200; BF9212, Affinity) overnight at 4 °C. Subsequently, the Cy3-conjugated goat anti-rabbit IgG antibody (1:200; A27039, Invitrogen, Carlsbad, CA, USA) or FITC-conjugated goat anti-mouse IgG antibody (1:200; Ab6785, Abcam, Cambridge, UK) was added and incubated for 90 min at room temperature.

To assess PASMC apoptosis in pulmonary arteries, paraffin-embedded sections were stained with an In Situ Cell Death Detection kit (12156792910, Roche, Basel, Switzerland) for 60 min at 37 °C. The α-SMA primary antibody (1:200) was used to stain smooth muscle cells in pulmonary arteries overnight at 4 °C, followed by FITC-conjugated goat anti-mouse IgG antibody (1:200) incubation for 60 min at room temperature.

For in vitro immunofluorescence staining, cells were incubated with primary antibodies against α-SMA (1:100), JMJD1C (1:100) or Ki67 (1:100) overnight at 4 °C, followed by Cy3-conjugated goat anti-rabbit IgG antibody (1:200) or FITC-conjugated goat anti-mouse IgG antibody (1:200) incubation for 60 min at room temperature. Cell nuclei were counterstained with DAPI. Images were observed under an Olympus microscope at 400× magnification.

### Lactate measurement

The lactate levels in serum or cell supernatants were measured by a lactate assay kit (A019, Jiancheng Bio, Nanjing, China) according to the manufacturer’s instructions.

### Quantitative Real-Time PCR (qPCR)

Total RNA was isolated from lung tissues and PASMCs using the TRIpure kit (RP1001, BioTeke, Beijing, China) and reverse-transcribed into cDNA by BeyoRT II M-MLV reverse transcriptase (D7160L, Beyotime, Shanghai, China). The primers for JMJD1C (forward 5’-GTTCACGGTCATTATACACG-3’, reverse 5’-AACCCTTCCTCTTATTTGG-3’), HK2 (forward 5’-CCCCTGCCACCAGACGAAA-3’, reverse 5’-CAGCCACAATGTCAATGTCAAAGTC-3’), PGK1 (forward 5’-AGCCAAGATTGTCAAAGA-3’, reverse 5’-TTCCCATTCAAATACCC-3’), and LDHA (forward 5’-CGGTTCCGTTACCTGAT-3’, reverse 5’-CTGTCCACCACCTGCTT-3’) were produced by Genscript (Nanjing, China). qPCR was performed using SYBR Green probes (SY1020, Solarbio) on an Exicycler^96^ Real-Time PCR detection system (Bioneer, Daejeon, Korea). The CT value for each sample was normalized to β-actin, and the relative expression was calculated by the 2^–△△CT^ method.

### Western blotting

Total protein lysates were extracted with cell lysis buffer (P0013, Beyotime) containing a protease inhibitor (ST506, Beyotime) and quantified with a BCA assay kit (P0011, Beyotime). Protein samples were loaded on SDS‒PAGE gels and transferred onto polyvinylidene difluoride membranes. After being blocked in 5% BSA for 1 h at room temperature, the membranes were hybridized with primary antibodies against JMJD1C (1:1000; A20153, ABclonal, Shanghai, China), HK2 (1:1000; A0994, ABclonal), PGK1 (1:1000; A12686, ABclonal), LDHA (1:1000; A0861, ABclonal), p-STAT3 (1:1000; AF3293, Affinity), STAT3 (1:1000; AF6294, Affinity), cyclin D1 (1:1000; A19038, ABclonal), CDK4 (1:1000; A11136, ABclonal) or β-actin (1:1000; sc-47778, Santa Cruz, Dallas, TX, USA) overnight at 4 °C, followed by goat anti-rabbit IgG (1:5000; A0208, Beyotime) or goat anti-mouse IgG (1:5000; A0216, Beyotime) secondary antibody incubation for 45 min at 37 °C. The protein signals were visualized by an enhanced chemiluminescence system (P0018, Beyotime) and quantified using Gel-Pro-Analyzer Software (WD-9413B, Beijing Liuyi Biotechnology Co., Ltd., Beijing, China).

### Statistical analysis

All results are expressed as the mean ± SD. Significant differences was analyzed using GraphPad Prism 8.0 Software (GraphPad Software Inc, La Jolla, CA, USA). Comparisons between two groups were analyzed by unpaired t test, and comparisons among three or more groups were analyzed by one-way ANOVA followed by Bonferroni’s multiple comparison test. A *p* value of less than 0.05 indicated significance.

## Supplementary information

Original Western blots

Reproducibility checklist

## Data Availability

Data that support the findings of this study are available from the corresponding author on reasonable request.
