# Supplementary figures and images for: JMJD1C promotes smooth muscle cell proliferation by activating glycolysis in pulmonary arterial hypertension
Source: Cell Death Discov. 2023 Mar 18;9:98. doi: 10.1038/s41420-023-01390-5 (PMC10024756; doi:10.1038/s41420-023-01390-5)

Figure 1 (D)

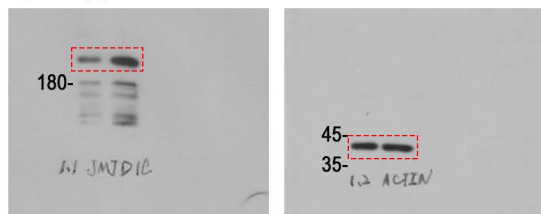

Figure 3 (B)

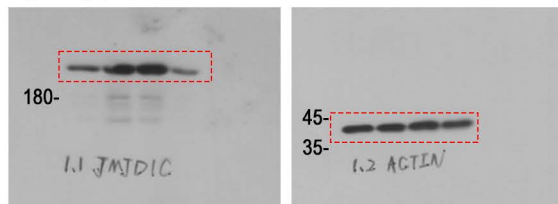

Figure 4 (B)

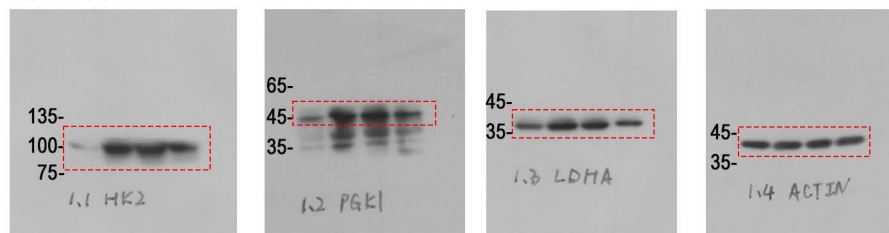

Figure 4 (D)

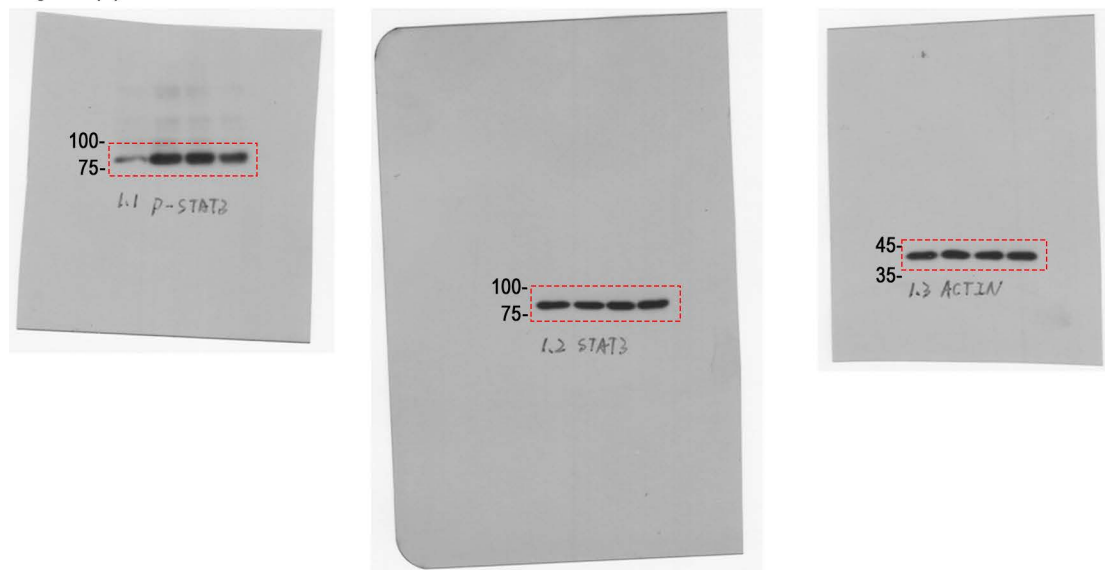

Figure 5 (C)

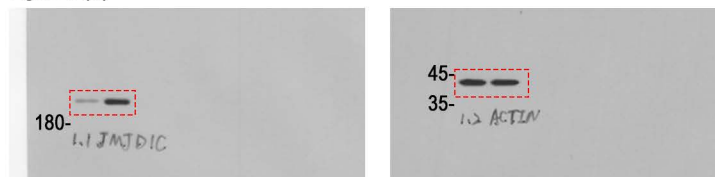

Figure 5 (F)

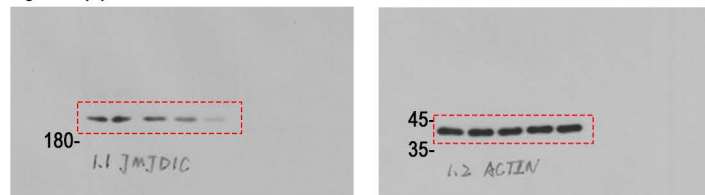

Figure 5 (H)

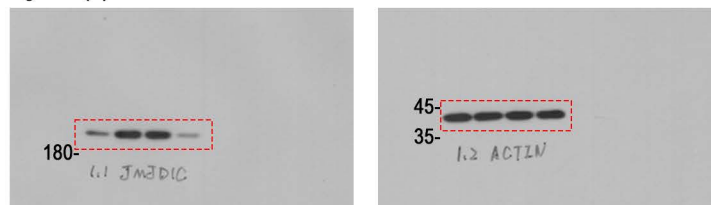

Figure 6 (C)

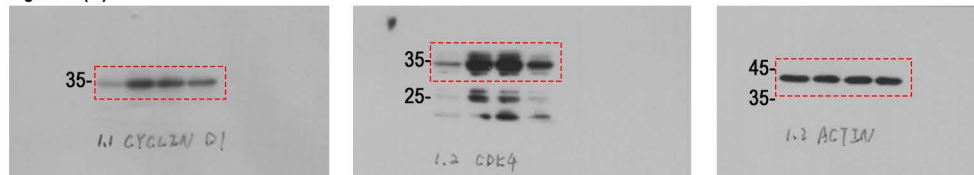

Figure 6 (E)

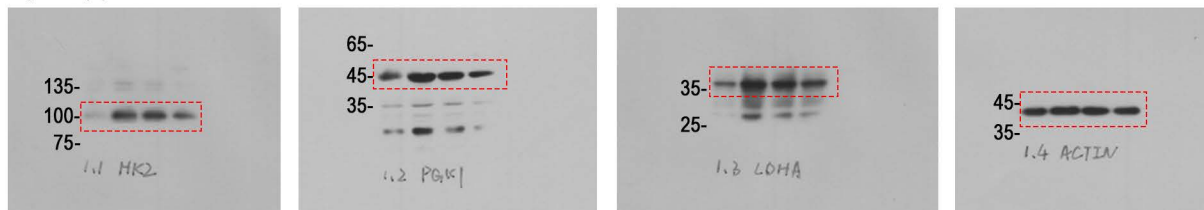

Figure 7 (A)

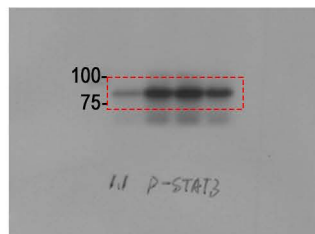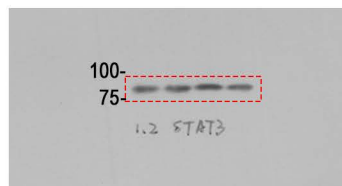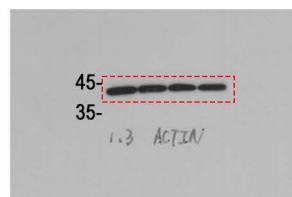

Figure 7 (D)

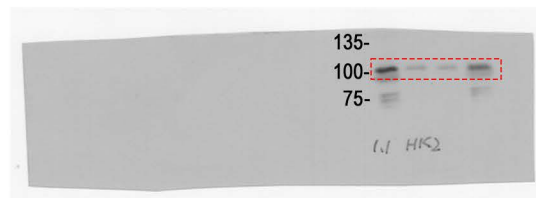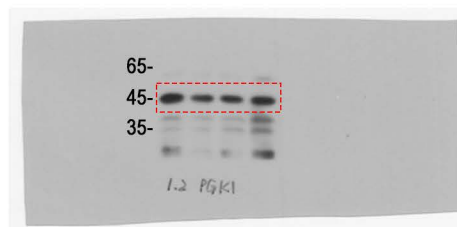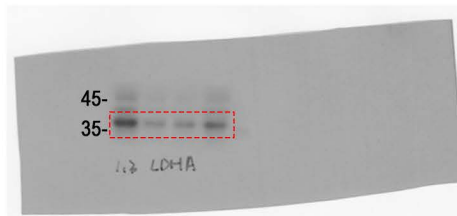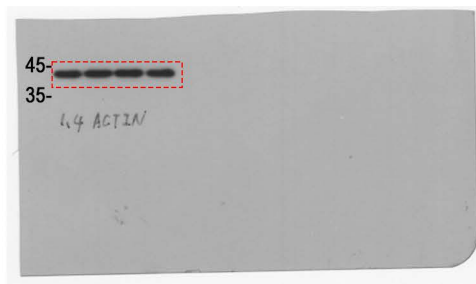

Supplement: Supplementary file 1 — Original Western blots [file 41420_2023_1390_MOESM1_ESM.pdf]
